# Methicillin-resistant *Staphylococcus aureus* has phenotypic variation in *mecA* expression that alters antibiotic sensitivity

**DOI:** 10.1128/aac.00396-25

**Published:** 2026-02-12

**Authors:** Dongzhu Ma, Rekha Arya, Beth Ann Knapick, Nadine M. Sadaka, Jewelia Rempuszewski, Claudette Moul, Jonathan B. Mandell, Neel B. Shah, James B. Doub, Charles G. Gish, Dana M. Parker, Niosha Parvizi, Stefanie A. Sydlik, Hunter B. Wood, Alecia B. Rokes, Vaughn S. Cooper, Anthony R. Richardson, Robert M. Q. Shanks, Kenneth L. Urish

**Affiliations:** 1 Bethel Musculoskeletal Research Center, Department of Orthopaedic Surgery, University of Pittsburgh 6614, Pittsburgh, Pennsylvania, USA; 2 Department of Ophthalmology, University of Pittsburgh 6614, Pittsburgh, Pennsylvania, USA; 3 Department of Internal Medicine, Division of Infectious Disease, University of Pittsburgh Medical Center 6595, Pittsburgh, Pennsylvania, USA; 4 Division of Clinical Care and Research, Institute of Human Virology, University of Maryland 1068 , Baltimore, Maryland, USA; 5 Department of Chemistry, Carnegie Mellon University 6612, Pittsburgh, Pennsylvania, USA; 6 Department of Microbiology and Molecular Genetics, University of Pittsburgh 6614, Pittsburgh, Pennsylvania, USA; 7 Arthritis and Arthroplasty Design Group, The Bone and Joint Center, Magee-Womens Hospital of the University of Pittsburgh Medical Center 6620 , Pittsburgh, Pennsylvania, USA; 8 Department of Bioengineering, and Clinical and Translational Science Institute, University of Pittsburgh 539033, Pittsburgh, Pennsylvania, USA; Shionogi Inc., Florham Park, New Jersey, USA

**Keywords:** methicillin-resistant *Staphylococcus aureus* (MRSA), β-lactam antibiotics, mecA, penicillin-binding protein 2a (PBP2a), rpoB, caseinolytic protease P (clpP), wall teichoic acid (WTA)

## Abstract

Methicillin resistant *Staphylococcus aureus* (MRSA) bacteremia has a high rate of morbidity and mortality. Multiple clinical studies have demonstrated improved outcomes when MRSA bacteremia is treated with dual antibiotic therapy that includes a β-lactam antibiotic such as cefazolin. This is a paradox as MRSA should be inherently resistant to this class of antibiotics. We report on a serendipitous observation of a phenotype where MRSA became sensitive to cefazolin when cultured in a physiologic relevant media of fetal bovine serum as well as in synovial fluid. This could be observed across multiple clinical isolates. Expected resistance was maintained when cultured in Muller Hinton Broth (MHB). MRSA β-lactam antibiotic resistance is mediated by PBP2a, a penicillin-binding protein encoded by *mecA*. We hypothesized that this phenotype of antibiotic sensitivity in physiologic medium was based, in part, on levels of PBP2a expression and post-translational modifications of peptidoglycan wall teichoic acid (WTA). We, therefore, conducted quantitative RT-PCR analysis and Western blotting which demonstrated limited *mecA* expression PBP2a protein level when cultured in FBS as compared to the clinical microbiology standard MHB, respectively. Whole genome sequencing of loss of function mutants generated through serial passaging in FBS revealed that the *clp* family of proteins and *rpo* genes were involved in β-lactam resistance. Cell wall peptidoglycan analysis suggested that WTA glycosylation was altered between β-lactam resistant and sensitive MRSA phenotypes. Together, this suggests that *clpP*, *rpoB,* and WTA glycosylation are involved with the β-lactam sensitivity phenotype in MRSA and can be new potential targets for MRSA treatment.

## INTRODUCTION

*Staphylococcus aureus* bacteremia has a high morbidity and mortality (1), but not all strains of *S. aureus* are the same. Outcome and treatment depend on the antibiotic resistance profile, with methicillin-resistant *Staphylococcus aureus* (MRSA) bacteremia being twice as likely to cause death than methicillin-susceptible *Staphylococcus aureus* (MSSA) bacteremia (2). The difference in antibiotic resistance between MRSA and MSSA to β-lactam antibiotics is conferred from the presence of the *mecA* gene (3, 4). It encodes a penicillin-binding protein (PBP2a) with low affinity for these antibiotics. Clinically, this resistance is determined primarily through the Clinical and Laboratory Standards Institute (CLSI) minimum inhibitory concentration assay using Mueller-Hinton Broth (MHB) (5). The class of antibiotic used to treat *S. aureus* infection is dependent on the results of this assay. Infectious Diseases Society of America (IDSA) guidelines recommend cefazolin, a first-generation cephalosporin as primary treatment for MSSA, and either daptomycin or vancomycin for MRSA (6).

However, multiple clinical studies have demonstrated possible therapeutic benefits and improved clinical outcomes in MRSA bacteremia when vancomycin or daptomycin was combined with a cephalosporin as compared to vancomycin or daptomycin monotherapy alone. A prospective randomized clinical study comparing dual therapy to standard of care monotherapy was halted as the monotherapy group had higher rates of mortality as compared to combination therapy (7). In a second randomized prospective study, dual therapy that included a first-generation cephalosporin decreased the overall time of bacteremia at the final measured time point as compared to monotherapy (8). The presence of an antibiotic resistance gene does not guarantee its expression, and there can be variation between an antibiotic resistance genotype and phenotype induced by different environmental conditions (9). Therefore, quantifying clinical antibiotic sensitivity using the CLSI protocol may fail to replicate the actual metabolic and environmental conditions of the infection leading to a variation between the actual and measured antibiotic resistance phenotype (10, 11).

Based on this and the unexpected benefit of dual therapy in MRSA bacteremia, we questioned if there was variability in the resistance phenotype of MRSA between physiologically relevant media and MHB, the media used in the CLSI protocol. We hypothesized that culturing MRSA in a physiologically relevant media would result in cefazolin sensitivity as compared to culturing in MHB. We demonstrated that MRSA cultured in MHB was resistant to β-lactam antibiotics but became sensitive to β-lactam antibiotics in a physiologically relevant medium of serum or synovial fluid. This phenotypic sensitivity was partially associated with a decrease in expression of PBP2a and post-translational modification of wall teichoic acid glycosylation.

## RESULTS

### MRSA strains become susceptible to beta-lactam antibiotic cefazolin when grown in FBS

**Fig 1:**
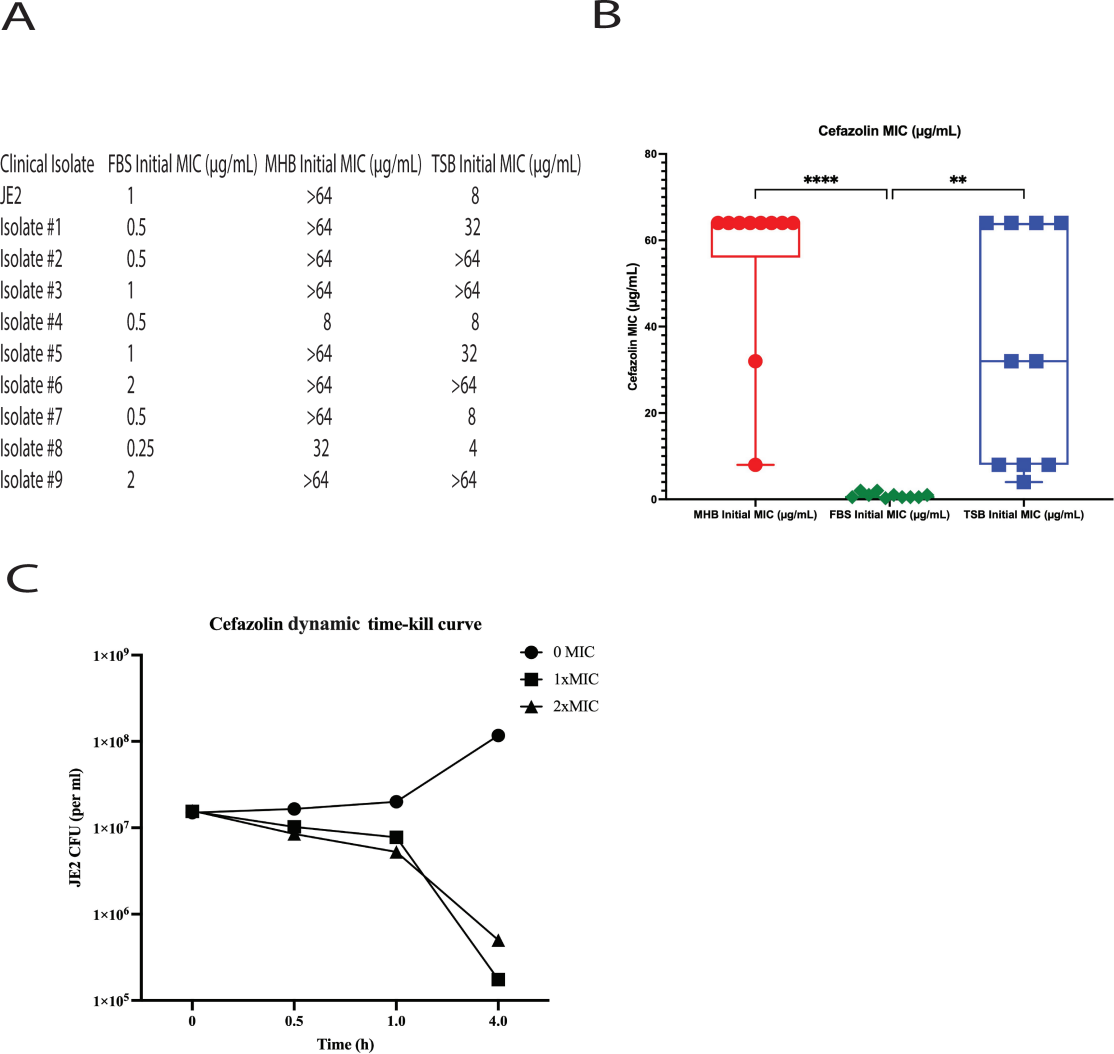
MRSA becomes susceptible to the beta-lactam antibiotic cefazolin when grown in FBS. (**A**) Cefazolin MIC assays were performed on JE2 and 9 clinical MRSA isolates in fetal bovine serum (FBS), Mueller Hinton Broth (MHB), and Tryptic Soy Broth (TSB), respectively. (**B**) MRSA isolates cultured in FBS were sensitive in cefazolin as compared to MRSA isolates cultured in MHB (****P < 0.0001) in TSB (**P < 0.01). Comparisons were performed using one-way ANOVA and Tukey’s multiple comparisons test. (**C**) JE2 dynamic kill-curve in FBS.

In a serendipitous observation, our group observed MRSA cefazolin resistance was a phenotype that was dependent on culture conditions. Resistance to cefazolin, a first-generation cephalosporin, as determined by MIC, was variable based on media type. MRSA laboratory strain (JE2) and 9 MRSA clinical isolates were resistant to cefazolin in MHB (greater than or equal to 8 µg/mL) (Fig. 1A), the culture media used in clinical microbiology laboratories. However, these isolates became sensitive (MIC<=2; Fig. 1B) to cefazolin in a culture media representing conditions similar to physiologic serum (fetal bovine serum, FBS). These experiments were repeated with cefaclor, a second-generation cephalosporin antibiotic, with phenotype similar to those of cefazolin (MIC = 16 µg/mL in TSB, >= 64 µg/mL in MHB, 4 µg/mL in FBS). Dynamic kill-curve assays demonstrated that the majority of bacteria was killed within 4 h (Fig. 1C). These observations are supported by the counterintuitive results of clinical studies that have demonstrated therapeutic benefit from dual antibiotic therapy with cefazolin in MRSA bacteremia (7, 8).

### MRSA isolates had decreased *mecA* mRNA in physiologic media that correlated with cefazolin sensitivity

To determine if this phenotype was observed in other physiologic environments, the experiment was repeated in an additional type of physiologic media, synovial fluid. Serum is not a defined media, but a defined version of synovial fluid termed pseudo synovial fluid (pSF) has been validated (12). This pSF is designed to replicate key characteristics of natural synovial fluid. When these same clinical isolates were cultured in pSF, the cefazolin MIC remained relatively low at 4 µg/mL.

**Fig 2:**
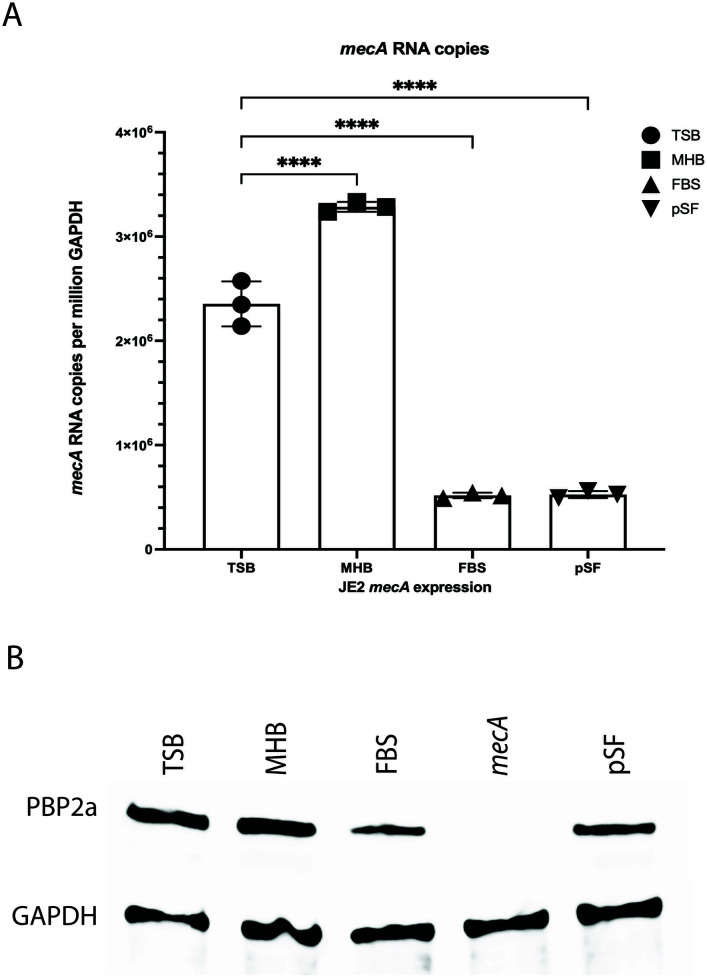
Physiologic culture media altered *mecA* mRNA and PBP2a expression. Quantitative real-time PCR and Western blotting were performed on JE2 cultured in TSB, MHB, FBS, and Pseudo synovial fluid (pSF). (**A**) MRSA (JE2) had higher levels of *mecA* mRNA when cultured in TSB and MHB as compared to FBS and pSF at a 4-h time point (****P < 0.0001). Comparisons were performed using one-way ANOVA and Dunnett’s multiple comparisons test. (**B**) MRSA (JE2) had higher levels of PBP2a when cultured in TSB and MHB as compared to FBS and pSF at a 4-hr time point. (*mecA* is a *mecA* gene mutation strain from NARSA library as a control).

To determine a mechanism for this phenotype that was observed in both serum and synovial fluid environments, we first wanted to determine if this was before or after translation of the primary gene associated with β-lactam resistance, *mecA*. We hypothesized that the phenotypic sensitivity to cefazolin was based on variable expression of the *mecA* gene. To test this hypothesis, we measured the expression levels of *mecA* (mRNA) and PBP2a, the protein encoded by the *mecA* gene, with quantitative PCR and Western blot, respectively, in different growth medium (Fig. 2). GAPDH was used as a reference control. *mecA* expression had a large and statistically significant reduction in FBS and pSF as compared to TSB and MHB (*P* < 0.0001, Fig. 2A). Protein levels of PBP2a were decreased in FBS and pSF media as compared in TSB and MHB determined by Western blot (Fig. 2B).

As studies have demonstrated that MRSA upregulates *mecA* expression in response to β-lactams (13), we measured *mecA* expression/PBP2a levels in FBS containing cefazolin. When MRSA was cultured in 1 × MIC cefazolin, *mecA* expression was downregulated (Fig. S1A *P* < 0.0001), but PBP2a levels remained comparable (Fig. S1C). *mecR1* is a *S. aureus* gene that plays a crucial role in regulating the expression of methicillin resistance. It responds to β-lactam antibiotics and triggers the activation of the *mecA* gene (14). We found that *mecR1* expression was downregulated (Fig. S1B, *P* < 0.001) in 1 × MIC cefazolin as well. In summary, these findings suggested that *S. aureus* JE2 *mecA* expression was decreased in physiologic media (FBS) as compared to standard laboratory media (MHB and TSB).

### JE2 resistant to cefazolin in physiologic media may be driven by *clp* and *rpo* genes

**Fig 3:**
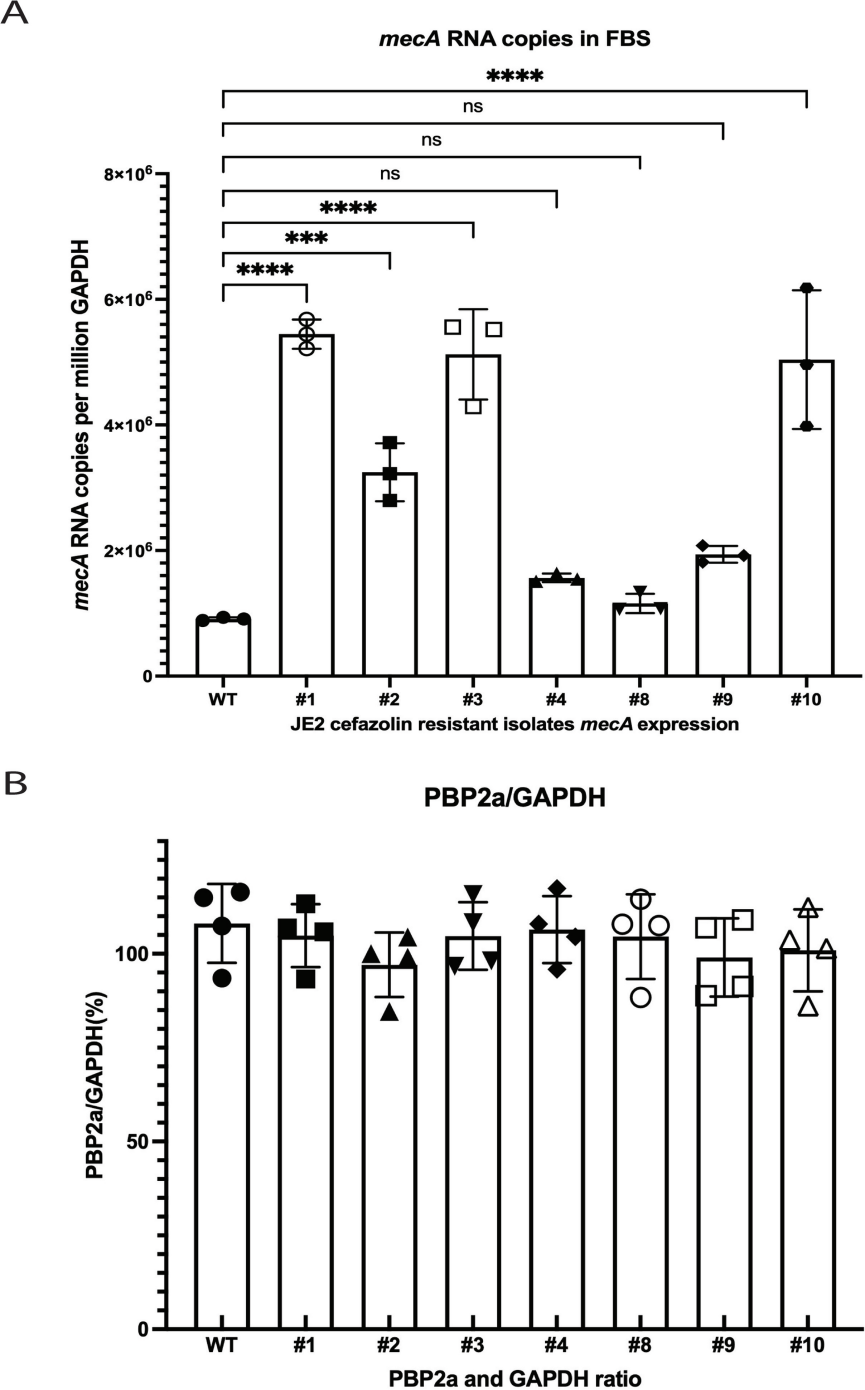
Serially passaged MRSA that has lost the phenotype of cefazolin sensitivity in physiologic media express more *mecA* mRNA than that of wild type in FBS. (**A**) Quantitative real-time PCR of these MRSA cefazolin resistant isolates in FBS was performed. These mutants expressed higher levels of *mecA* mRNA cultured in FBS compared with wild type. Comparisons were performed using one-way ANOVA and Dunnett’s multiple comparisons test (ns, no significance, ***P < 0.001, ****P < 0.0001). (**B**) The western blot was repeated four times and the band intensity measured relative to the GAPDH control band by densitometry. Comparisons were performed using one-way ANOVA and no appreciable differences in PBP2a levels between wild type and mutants were observed (*P* = 0.7465).

To identify possible genes responsible for this phenotypic cefazolin sensitivity, MRSA (JE2) was serially passaged in FBS until the cefazolin sensitivity phenotype was lost. We obtained 12 isolates that were initially sensitive to cefazolin in physiologic medium, lost the phenotype, and developed comparable resistance to cefazolin in physiologic and laboratory media (Table 1). Similar to the changes observed when MRSA is grown in physiological or laboratory media, the loss of cefazolin sensitivity in these mutants was associated with higher levels of *mecA* expression compared with the wild-type strain (Fig. 3A). However, we did not observe any significant changes in PBP2a levels in the resistant mutants compared to the sensitive wild type (Fig. 3B**,** *P* = 0.7465). Based on these findings, we speculate that the lack of changes in PBP2a levels suggests that other mechanisms affecting the distribution, modification, degradation, or conformational changes of PBP2a may be involved.

**TABLE 1:** JE2 cefazolin resistant isolates MIC in FBS (µg/mL)

Isolates	FBS initial MIC (µg/mL)	FBS final MIC (µg/mL)	MHB initial MIC (µg/mL)	MHB final MIC (µg/mL)	Passages
JE2	1	n/a	>64	>64	n/a^*a*^
Isolate #1		>16		>64	7
Isolate #2		>16		>64	9
Isolate #3		>16		>64	12
Isolate #4		>16		>64	9
Isolate #5		>64		>64	13
Isolate #6		>64		>64	8
Isolate #7		>64		>64	8
Isolate #8		>64		>64	8
Isolate #9		>64		>64	8
Isolate #10		>64		>64	9
Isolate #11		>64		>64	6
Isolate #12		>64		>64	11

^*a*^ n/a, not available.

Whole genome sequencing (WGS) was completed to identify the mutations that were associated with the loss of the phenotype (Table 2). Our genomic analysis revealed distinct mutations in either *rpo* or *clp* genes in serially passaged loss of function mutants. For the *rpo* family, there were seven isolates with missense mutations in the *rpoB* gene, one isolate with a missense mutation in the *rpoC* gene, and two isolates with a missense mutation in the *rpoD* gene. For the *clp* family, three isolates had missense mutations in the *clpP* gene, one isolate had a missense mutation in the *clpX* gene, and one isolate had a *clpX* frame shift mutation (−1bp). Mutations in *rpoB/rpoC*, encoding the β and β′ subunits of the RNA polymerase, can facilitate adaptation to diverse environmental, antibiotic stresses, and are responsible for resistance phenotypes (15–17). *rpoB* and *rpoC* have been reported to be involved in bacteria β-lactam resistance (17–19). The CLP (caseinolytic protease) family is linked to antibiotic resistance because these proteases are essential for bacterial survival under stress. They are crucial for stress response (20), regulating virulence and biofilm formation (21), involving in antibiotic resistance (22), and have been identified as a potential therapeutic target (23).

**TABLE 2:** NGS genomic DNA analysis of Cefazolin resistant isolates

JE2 resistant isolates	Gene name	Nucleotide position of mutation	Nucleotide change	Change to coding sequence
#1	*rpoD*	1672974	C->A	G320C
#2	*rpoC* *clpX*	595332 1777212	C->A Deletion (−1bp)	A6D Shifted
#3	*clpP*	853541	G->T	D27Y
#4	*rpoB*	594491	C->A	S964Y
#5	*rpoB*	592755	T->A	S385R
#6	*rpoB* *clpP* *pyrR*	593825 853968 1208866	C->A C->A G->C	A742E T169K D145H
#7	*rpoB* *purK* *sfaA* *cntA* *icaB*	593585 1074893 2316025 2597305 2829450	A->A T->C T->C T->C A->T	E662V M31T Y62C Y379C E83D
#8	*clpP* *guaA*	853544 440603	C->T A->C	R28C T178P
#9	*rpoB* *clpX*	592754 1776459	G->T A->G	S385I L335P
#10	*rpoB*	592295	C->A	A232E
#11	*rpoD* *guaA* *gatC* *wecB*	1672970 441605 2045663 2229298	A->G T->C T->C T->C	L321P W512R H55R I250V
*#12*	*rpoB*	595031	T->A	V1144D

Based on the above, we hypothesized that these mutations in *rpo* or *clp* may play a role in cefazolin resistance. Wall teichoic acid glycosylation has also been demonstrated to alter cefazolin sensitivity in MRSA (24, 25). To test this hypothesis, we measured the MIC of available *clp* and *rpo* transposon mutant strains that had been verified with PCR (*clp, clpB, clpC, clpP, rpoE, rpoF;* NARSA library). We also tested WTA transposon mutant strains *tarM* and *tarS* (NARSA library) and deletion mutant strains *tarO* (26) and *tarP* (27) (Table 3). Similar to the *clpP* loss of function mutant identified in the serial passage experiments (isolate #3), the *clpP* transposon mutant from the NARSA library displayed resistance to cefazolin in FBS, in contrast to the wild-type control strain. This finding indicates that clpP contributes to overall cefazolin susceptibility rather than specifically mediating susceptibility in the presence of FBS, suggesting that *clpP* is involved with cefazolin resistance.

**TABLE 3:** Cefazolin MIC assays of NARSA mutant and tar mutant strains (µg/mL)

Strains	WT (JE2)	*clp^a^*	*clpB*	*clpC*	*clpP*	Resistant isolate #3 (*clpP*)	*rpoE*	*rpoF*	*tarM*	*tarO^b^*	*tarP^c^*	*tarS*
TSB	8	8	8	8	32	≥64	8	2	8	4	8	8
MHB	≥64	≥64	≥64	≥64	≥64	≥64	32	4	≥64	32	≥64	≥64
FBS	1	1	1	1	8	32	2	1	1	0.25	1	1

^*a*^ Strain Clp, clpB, clpC, clpP, rpoE, rpoF, TarM, and TarS are from NARSA library.

^*b*^ Strain TarO is from Dr. Suzanne Walker’s lab (26).

^*c*^ Strain TarP is from Dr. Andreas Peschel’s lab (27).

**Fig 4:**
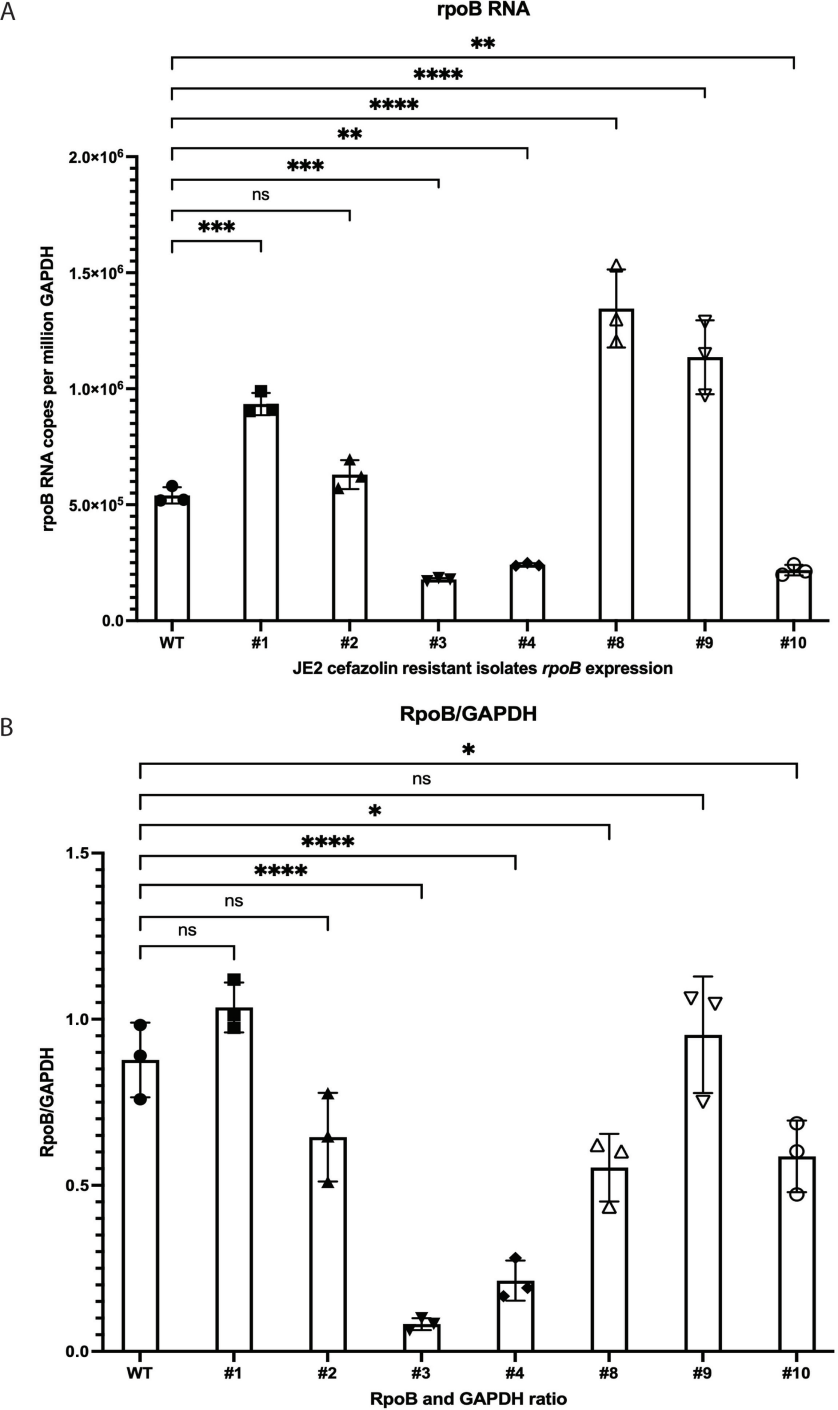
*rpoB* RNA levels and RpoB protein levels of cefazolin resistant isolates in FBS. (**A**) *rpoB* RNA levels of JE2 cefazolin resistant isolates in FBS. (**B**) RpoB protein levels of JE2 cefazolin resistant isolates in FBS. The Western blot was repeated three times and the band intensity measured relative to the GAPDH control band by densitometry. Comparisons were performed using one-way ANOVA and Dunnett’s multiple comparisons test (ns = no significance, *P < 0.05, **P < 0.01, ***P < 0.001, ****P < 0.0001).

In our genomic analysis, the majority of mutations occurred in the *rpo* family of genes, and so we attempted to understand the role of *rpoB* in mediating cefazolin resistance in physiologic media. *rpoB* RNA levels (Fig. 4A) and RpoB protein levels (Fig. 4B) were measured in each serially passaged isolate and were found to be different in each isolate, suggesting that expression levels may not be responsible for this β-lactam resistance phenotype in physiological medium. Using the NARSA library’s available *rpo* and *clp* mutants (*clp, clpB, clpC, clpP, rpoE, rpoF*, and *mecA*), we then determined if the transposon insertions in these genes would lead to alteration of RpoB expression. We did not observe any significant differences in RpoB protein levels between the *clp* and *rpo* mutants (Fig. S2). Finally, to test if the loss of RpoB could lead to β-lactam resistance, we attempted to create a *rpoB* gene deletion mutant by homologous double crossover recombination using pKFT-rpoB vector (28) (Supplemental data). This was attempted more than four times and was not successful. The failure to generate a viable *rpoB* deletion mutant strongly suggests that RpoB is essential for the survival of *S. aureus* JE2. Overall, these findings suggested that the RpoB protein level was not associated with cefazolin resistance and was more likely attributed to an alternative mechanism such as conformational or functional change.

### Glycosylation of cell wall teichoic acids is altered in FBS as compared to MHB

**Fig 5:**
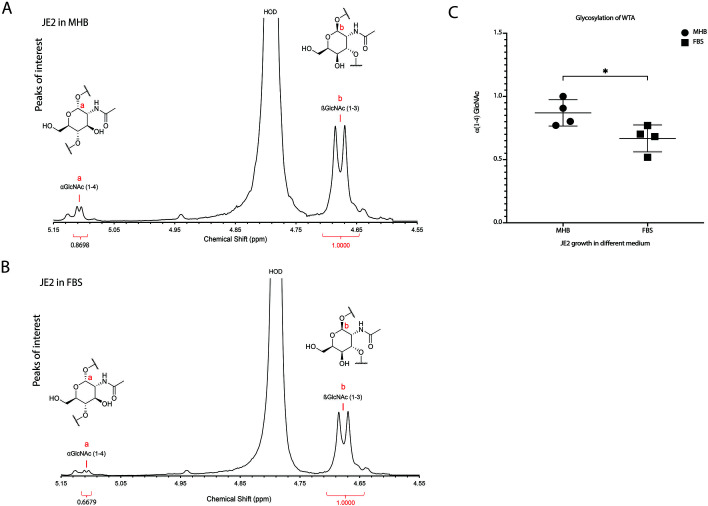
Glycosylation of JE2 cell wall teichoic acids (WTA) decreased in FBS than that in MHB. (**A**) JE2 strain α (1–4) GlcNAc of WTA mean is 0.8698, β (1–3) GlcNAc of WTA is 1.0 in MHB. (**B**) JE2 strain α (1–4) GlcNAc of WTA mean is 0.6679, β (1–3) GlcNAc of WTA is 1.0 in FBS. (**C**) α (1–4) GlcNAc of WTA decreased in FBS than that in MHB (*P* = 0.0353). Comparisons were performed using unpaired *t*-test (*P < 0.05).

Cell wall teichoic acids (WTA) play a crucial role in the structural integrity and pathogenicity of *S. aureus*. Different glycosylation patterns of WTA have been demonstrated to alter β-lactam sensitivity in MRSA (24, 25). Furthermore, environmental conditions, such as the nutrient composition of the growth medium, can affect WTA modifications (24). As a result, we hypothesized that WTA glycosylation patterns may be altered in physiologic growth medium as compared to standard laboratory medium. To assess this, we compared WTA glycosylation patterns in JE2 cultured in MHB and FBS, using the ratio of peaks at 5.1 ppm and 4.7 ppm corresponding to the α (1–4) GlcNAc proton of WTA and the β (1–3) GlcNAc proton of WTA, respectively. Proton nuclear magnetic resonance (NMR) spectra demonstrated the α (1–4) GlcNAc mean was 0.870 in MHB and 0.670 in FBS (*P* = 0.035), confirming that different culture media (MHB vs FBS) induced different patterns of WTA glycosylation (Fig. 5). We also collected several JE2 WTA glycosylation mutants (*tar* mutants) (*tarM, tarO, tarP, tarS*) and tested the cefazolin MIC in TSB, MHB, and FBS. The cefazolin MICs of *tarM, tarP,* and *tarS* mutants were identical to those of the JE2 wild-type strain. The cefazolin MIC of *tarO* mutant was more sensitive than WT (Table 3). Overall, these results concluded that the glycosylation pattern of WTA may contribute to increased JE2 cefazolin susceptibility in FBS.

## DISCUSSION

Methicillin-resistant *Staphylococcus aureus* (MRSA) infections can have high morbidity and mortality.

Resistance to β-lactam antibiotics is determined by the *mecA* gene, which encodes the penicillin-binding protein PBP2a. Penicillin-binding proteins (PBPs) are a class of proteins that are essential for cell wall synthesis and can lead to cell lysis and death (24). Methicillin-resistant *Staphylococcus aureus* (MRSA) acquires resistance to β-lactam antibiotics through the *mecA* gene, allowing cell wall synthesis to continue.

A series of clinical studies have observed the paradoxical observation that the addition of a first-generation cephalosporin to vancomycin or daptomycin can improve clinical outcomes. This is unexpected as MRSA is resistant to these first-generation cephalosporins. An initial pilot study observed a decrease in bacteremia from 3.0 days in the monotherapy group (daptomycin or vancomycin) to 1.9 days in the dual therapy group that included flucloxacillin (*P* = 0.06) (29). This led to a randomized open label clinical study that compared standard monotherapy (vancomycin or daptomycin) to dual therapy with vancomycin/daptomycin and a series of similar β-lactam antibiotics (cefazolin, cloxacillin, flucloxacillin) (8). The study was discontinued early as increased renal toxicity was observed in the dual therapy group, and the initial primary outcome of mortality was unable to be observed. The authors speculated that if they limited the use of the β-lactam antibiotic to cefazolin which has a better safety profile, this early stopping criteria would have possibly been avoided. Comparable to the initial study, they did observe a statistically reduced time period of bacteremia at the final time point at day 5 in the dual therapy group (*P* = 0.02). This is relevant as bacteremia increase the probability of seeding additional organ systems that often require surgical intervention. Clinically many physicians used this early data to support the use of dual therapy in *S. aureus* bacteremia with the selection of a cephalosporin that had measured MIC activity using a standard CLSI protocol (7, 30).

Here, we observed MRSA antibiotic resistance to β-lactam antibiotics can be variable. MRSA resistance to β-lactam was altered when cultured in physiologic medium (FBS or synovial fluid) as compared to traditional laboratory medium (TSB or MHB). In physiologic culture medium, MRSA clinical isolates developed a phenotype that was sensitive to β-lactam antibiotics and reverted when cultured in MHB, the media used to clinically determine resistance. This sensitivity was based, in part, on limited *mecA* expression levels, decreased PBP2a protein levels, and WTA post-translational modifications.

Based on the characteristics of PBPs in antibiotic resistance, we assessed cefazolin resistance by measuring *mecA* expression and PBP2a level. Compared to cultures in MHB, *mecA* mRNA and PBP2a expression levels were reduced in FBS (Fig. 2). We hypothesized that environmental factors present in host conditions of *S. aureus* infections could influence *mecA* expression thereby impacting antibiotic efficacy. Pseudo synovial fluid (pSF) was used to mimic the *S. aureus* host joint microenvironments. Our results demonstrated that antibiotic susceptibility could vary depending on the culture medium. Specifically, JE2 exhibited increased susceptibility to cefazolin in pSF and FBS, compared to standard laboratory media MHB. This may be a result of decreased *mecA* expression and subsequent levels of PBP2a.

To determine a mechanism driving the change in resistance, WGS identified that RpoB played an important role in the phenotype. The *rpoB* gene encodes the β subunit of bacterial RNA polymerase. Mutations in *rpoB* can offer resistance to rifampicin because they alter the drug-binding residues of the protein, thereby reducing affinity for these antibiotics (31–33). Further studies showed that the *rpoB* mutation resulted in decreased expression levels of anaerobic respiration and fermentation genes, while 11 genes such as *mecA* were specifically upregulated (17). This may connect RpoB with β-lactam antibiotic resistance. When bacteria are placed under diverse selective pressures, they often adapt rapidly by mutating to the most conserved positions in *rpoB* (16). In this study, JE2 cefazolin resistant isolates in FBS were obtained by serial passages. Genomic DNA analysis of these isolates revealed that *rpoB* was most likely involved in the loss of phenotype and developing resistance to cefazolin. However, the exact mechanism of resistance remains unknown. Changes in RpoB conformation or other mechanisms may be involved, such as reduced cell wall permeability, cell wall thickness, or enhanced efflux pumps.

Peptidoglycan (PG) is the major structural component of the bacterial cell wall. Gram-positive bacteria are characterized by a thick cell wall composed of a peptidoglycan matrix embedded in anionic polyol phosphate polymers, known as teichoic acids (34–36). WTA is a major component of the *S. aureus* cell wall and plays an important role in the bacteria’s physiology (adhesion, colonization, and cell division) (37), virulence, biofilm formation, and resistance to antimicrobial molecules (38, 39) and is covalently attached to peptidoglycan via a phosphodiester linkage (40). WTA glycosylation is influenced by environmental conditions and contributes to resistance to antibiotics (24). Glycosyltransferases TarS and TarM are involved in WTA α-O-GlcNAcylation and β-O-GlcNAcylation, respectively, and are essential for normal cell function (24). We observed that culturing JE2 in a physiologic media altered its WTA glycosylation (α (1–4) GlcNAc). This suggests that WTA post-translational processes may also play a role in this observed β-lactam resensitization.

While these findings provide a mechanistic underpinning for the potential benefit of dual therapy for MRSA bacteremia, there are limitations in this study. Only 9 clinical MRSA strains and 12 JE2 cefazolin-resistant isolates were analyzed, as well the number of isolates containing certain types of *rpoB* mutations was still limited. Because *rpoB* is a gene essential for bacterial survival, manipulation of the *rpoB* gene can easily lead to lethal consequences. Therefore, it was difficult to conduct mutagenesis studies on *rpoB* genome manipulation. However, it will be crucial to introduce the specific *rpoB* mutations discovered by whole-genome sequencing into a background of cefazolin-susceptible MRSA to establish causality. This remains an important experiment for our future studies.

### Conclusion

The expression of *mecA* and PBP2a has been extensively studied in relation to methicillin (oxacillin) resistance. We observed that MRSA could become sensitive to β-lactam antibiotics (cefazolin) when cultured in a physiologically relevant media. This phenotype was partially associated with *mecA* and PBP2a protein expression. *rpoB* and *clpP* genes were associated with this phenotype. This identifies a potential new therapeutic target for MRSA infections and suggests a new strategy for treatment of other multidrug resistant infections.

## MATERIALS AND METHODS

### Bacterial strains, plasmids, and growth conditions

The bacterial USA300 FPR3757 strain (BAA-1556, JE2) was purchased from The American Type Culture Collection (ATCC). NR-48501 *Staphylococcus aureus* subsp. aureus Nebraska Transposon Mutant Library (NARSA) was obtained from BEI resources (https://www.beiresources.org/Home.aspx). Trypticase soy broth (TSB) and Mueller–Hinton II broth (MHB) were purchased from BD (USA) and sterilized by autoclaving. Fetal bovine serum (FBS) was purchased from Invitrogen (Thermo Fisher Scientific, USA). *Staphylococcus aureus* strains were grown in TSB medium with or without appropriate antibiotics. Ampicillin, Erythromycin, Tetracycline, Chloramphenicol, cefazolin, Vancomycin, Rifampin, Lysostaphin, Anti-RpoB, and Anti-PBP2a of MRSA were purchased from Sigma-Aldrich (USA). Anti-GAPDH (GA1R) antibody and goat anti-mouse IgG (H+L) secondary antibody-HRP were purchased from Invitrogen (Thermo Fisher Scientific, USA). SDS-PAGE and protein electrophoresis system and ECL reagents were purchased from Bio-Rad (USA). iBlot3 protein semi-dry transfer system was purchased from Invitrogen (Thermo Fisher Scientific, USA).

### Minimum inhibitory concentration assay

The minimum inhibitory concentration (MIC) of cefazolin, cefaclor, or other antibiotics for *S. aureus* JE2 in suspension was determined using CLSI assay protocols. Briefly, one colony of bacteria was inoculated into 4 mL fresh TSB and cultured overnight. The cultures were diluted to 5 × 10^8^ CFU/mL concentration. Each well was inoculated with 0.5 × 10^6^ CFU of bacteria in different culture media (TSB, MHB, FBS, or pSF). Serial 1:2 dilutions of each antimicrobial working stocks were prepared by dissolving the antimicrobial stock solution in an appropriate solvent. Final antibiotics concentrations ranged 0, 0.25, 0.5, 1, 2, 4, 8, 16, 32, 64 µg/mL. Plates were placed in an incubator at 37°C for 24 h. Inhibition of bacterial growth in treated cultures was observed by turbidity assay using a SynTek microplate reader measuring absorbance at 600 nm (Thermo Fisher Scientific, USA).

### Dynamic time-kill curve

JE2 strain was prepared as the MIC assay above. A volume of 100 μL FBS diluted bacteria per well was transferred to 96-well plates (1.5 × 10^7^ CFU). One hundred microliters of FBS containing 0×MIC, 1×MIC, 2×MIC cefazolin was added, respectively.

Bacteria were grown shaking at 250 rpm at 37°C. Bacterial growth was monitored over a time-course of 4 h (0, 0.5, 1, 4 h). For every sampled time point, the content of three wells was removed and CFU assays were performed. Growth curves were analyzed by plotting the log CFU/mL against the time.

### Pseudo synovial fluid preparation

Pseudo synovial fluid (pSF) was prepared by combining 3 mg/mL Hyaluronic Acid (HA), 9 mg/mL Fibrinogen (Fg), and 10 mg/mL Albumin (Alb) in Dulbecco’s modified Eagle medium supplemented with 5% Non-Essential Amino Acids (DMEM) (12). Each component was also tested individually at the stated concentration in DMEM. MIC of JE2 was assessed as described above.

### Serial passage and cefazolin resistant isolates selection

Using a well-characterized MRSA strain, USA300 FPR3757 strain (BAA-1556, ATCC), we selected for cefazolin resistance by serial passage. A well-isolated colony was picked from an overnight agar plate and inoculated into TSB medium to achieve the specified turbidity by comparing to a 0.5× McFarland turbidity standard (1 × 10^8^/mL). Approximately 1 × 10^6^ cells were added to each well of a 96-well plate that was filled with FBS containing half diluted cefazolin. The plates were incubated overnight at 37°C. We recorded antibiotic concentration where bacteria growth was inhibited (MIC). The wells just below the observed MIC were mixed and then pipetted into a 2 mL centrifuge tube. The harvested bacteria were replated into fresh medium containing the cefazolin, and MICs were repeated until the cefazolin MIC in FBS reached ≥64 μg/mL. Next, the ≥64 μg/mL well was transferred into a sterile tube and mixed. Using an inoculating loop, a streak plate was performed and then incubated overnight at 37°C. The streak plate was passaged for 10 days. After 10 days, culture clones were grown overnight in 4 mL of TSB at 37°C. The MIC assay was repeated with FBS. If the plate reached an MIC >16 µg/mL, the isolates were banked and then stored at −80°C.

### Genomic bacterial DNA isolation

The method for isolating genomic bacterial DNA is described in the supplemental data.

### Bacterial whole genome sequencing and variant calling

Sequencing libraries were prepared using the SeqWell purePlex kit (Beverly, MA). All samples were sequenced to at least 50-fold average coverage using an Illumina NextSeq500. Reads were trimmed and filtered for quality using Trimmomatic version 0.36 (41). Variants were identified by mapping reads from putative mutants against the *S. aureus* JE2 reference genome using breseq version 0.36 (42). Mutation calls were filtered to remove false positives due to universal differences between the founding isolate and the JE2 reference.

### Preparation RNA standards for JE2 *S. aureus mecA* gene

The standard curve method was selected to quantify *mecA* mRNA copies. To obtain RNA standards, the *mecA* gene fragments were amplified from JE2 *S. aureus* strain. The primers for *mecA* and GAPDH genes were JE2-mecA-standard-out-F (5′-TTTgaattcCCGTTCTCATATAGCTCATCATACA-3′), JE2-mecA-standard-out-R (5′-ATTggatccTGTTGGTCCCATTAACTCTGAA-3′). JE2-GAPDH-standard-out-F (5′-AGGTgaattcGAGGTAGTTGATGGTGGTTTC-3′), JE2-GAPDH-standard-out-R (5′- ATGggatccCGTTATCATACCAAGCTGCAAC-3′). The fragment was cloned into pBlueScript II SK+ (Agilent Technologies, USA) and linearized by *XbaI* as a DNA template for RNA *in vitro* transcription. Standard RNA template was produced by using Ambion MEGAscript kit (Thermo Fisher Scientific, USA) following kit manual instruction. Standard RNA concentration was determined by NanoDrop 2000 Spectrophotometers (Thermo Fisher Scientific, USA).

### Isolation of RNA and quantitative RT-PCR analysis

RNA isolation and quantitative reverse transcription polymerase chain reaction (RT-PCR) were performed by following the manufacturer’s instruction of the product. *S. aureus* was grown in 4 mL of TSB medium supplemented with appropriate antibiotics overnight at 37°C (about 16 h). Two hundred microliters of overnight growth was added into 4.0 mL fresh TSB medium and then grown at 37°C for 2 h. All 4 mL of bacterial culture was pelleted. One hundred microliters of TE Buffer was added to resuspend the cell pellet. Five microliters of Lysostaphin (5 mg/mL) was added to the resuspended pellet of bacteria. The samples were placed at 37°C for 15 min to destroy the cell wall. Total RNA was extracted by using TRIzol Max Bacterial RNA Isolation Kit (Thermo Fisher Scientific), and reverse transcription of RNA to single-stranded cDNA was performed using SuperScript IV Reverse Transcriptase (Thermo Fisher Scientific, USA). The newly synthesized cDNA was used immediately or frozen at −80°C.

Quantitative RT-PCR analysis was performed by using the CFX96 Real-Time System (BioRad, USA) and TaqMan Fast Advanced Master mix (Thermo Fisher Scientific, USA). The cycling conditions used were initial cycle 95°C for 2 min, followed by 45 cycles of 95°C for 10 s and 60°C for 60 s. The primers for *mecA* and *GAPDH* genes were JE2-mecA-qPCR-F (5′- TTGTAGCTAGCCATTCCTTTATCT-3′), JE2-mecA-qPCR-Probe (5′- ACCACCCAATTTGTCTGCCAGTTTC-3′), JE2-mecA-qPCR-R (5′- CCGGTACTGCAGAACTCAAA-3′). JE2-GAPDH-qPCR-F (5′-CACCAAGAGTTAGACGGTTCTG-3′), JE2-GAPDH-qPCR-Probe (5′-TGACTACAATTCACGCTTACACAGGTGA-3′), JE2-GAPDH-qPCR-R (5′- TGAGGTGCGTCTTGTGTATTT-3′). The actual RNA copies were calculated according to the standard curve. Statistical analysis was performed by using one-way ANOVA.

### Detection of PBP2a and RpoB levels by western blot

Overnight-grown *S. aureus* cells were inoculated at a 1:100 dilution in 4 mL of growth medium and grown at 37°C for 2–4 h. One thousand five hundred microliters of *S. aureus* cells was collected by centrifugation at 5,000 × *g* for 10 min at 4°C. The cell pellet was washed twice with 1.5 mL cold PBS and spun as before. The cell pellet was resuspended in 150 μL of TE buffer by vigorous vertexing. Five microliters of lysostaphin (5 mg/mL) was added to the cell resuspension and incubated at 37°C for 15–30 min, and the tube was inverted several times until the mixture became very viscous and transparent. The samples were sonicated on ice for 15 s. One hundred microliters of of Lysis buffer (50 mM Tris-HCl, 7.5 pH, 1% Triton X-100, 20 μg/mL DNase, 10 μg/mL RNase, 1× protease inhibitor cocktail) was added to the mixture and then incubated on ice for 30 min. Cells were centrifuged at 12,000 × *g* for 10 min at 4°C. The supernatant was retained. The protein concentration of the lysate was determined using the Bradford assay (Bio-Rad), and all samples were adjusted to the same protein concentration. Ten micrograms of total protein was separated using SDS-PAGE with Mini-PROTEAN precast gels (Bio-Rad). Proteins were transferred to a PVDF membrane using iBlot 3 Western Blot Transfer system (Thermo Fisher Scientific). The PVDF membrane was blocked with EveryBlot Blocking Buffer (Bio-Rad) at room temperature for 2 h. Primary antibodies against PBP2a, RpoB, or GAPDH (diluted 1:2,000 in EveryBlot blocking buffer) were applied to the membrane and shaken overnight in the cold room. A horseradish peroxidase (HRP)-conjugated goat anti-mouse IgG secondary antibody (Thermo Fisher Scientific) (diluted 1:5,000 in EveryBlot blocking buffer) was incubated with the membrane for 2 h. Proteins were visualized using Clarity ECL Western Blotting Substrate (Bio-Rad). Chemiluminescence detection was performed using the ChemiDoc MP system (Bio-Rad). Quantitative analysis of Western blots was performed using the free software Fiji: ImageJ (43).

### Extraction, purification, and analysis of bacterial wall teichoic acids

WTAs were extracted and purified from the JE2 strains grown in 200 mL culture of MHB and FBS for 24 h at 37°C. Overnight cultures were centrifuged and washed with Buffer1 (50 mM MES, pH 6.5). The cultures were inactivated by treatment with phenol-ethanol (1:1, vol/vol) to a final concentration of 2%. The cells were collected by centrifugation at 2,500*g* for 1 h at 4°C and suspended in 0.05 M Tris, 2 mM MgSO_4_, pH 7.5 (0.5 g wet weight/mL). The cell suspensions were incubated with lysostaphin (50 μg/mL) at 37°C for 3 h with continuous stirring. MgCl_2_ and Benzonase were subsequently added to a final concentration of 1 mM and 50 UI/mL, respectively, and incubated at 37°C for 4 h. The final concentration of Tris buffer was adjusted to 50 mM, and CaCl_2_ and Pronase were added to a final concentration of 1 mM and 0.5 mg/mL, respectively. Samples were incubated for 16 h at 37°C. The remaining insoluble cell debris was removed by centrifugation at 8,000*g* for 30 min. The supernatants were precipitated with 25% ethanol in the presence of 10 mM CaCl_2_ and stirred for 16 h at 4°C. The precipitates were removed by centrifugation at 8,000*g* for 30 min. The supernatants containing WTAs were precipitated with 75% ethanol in the presence of 10 mM CaCl_2_ and stirred for 4 h at 4°C. Then, WTAs were collected by centrifugation at 8,000*g* for 30 min and dissolved in water. The samples were dialyzed extensively against water at room temperature. After dialysis for 24–48 h, the residues were lyophilized, and the solids were frozen and kept in −80°C freezer. The breakdown of glycosylation states of the WTA of each sample were analyzed by Dr. Sydlik and Dr. Hunter Wood from Carnegie Mellon University.

### Proton nuclear magnetic resonance WTA analysis

WTA glycosylation states were determined using proton nuclear magnetic resonance spectroscopy (1H NMR). Lyophilized samples were blanketed with nitrogen to discourage proton exchange between the deuterium oxide (D2O) solvent and atmospheric water. Samples were then dissolved in D2O (0.7 mL, Cambridge Isotope Laboratories, Inc.) and mixed with a centrifuge vortex mixer to encourage sample dissolution. The dissolved samples were then transferred to NMR tubes and blanketed with nitrogen. 1H NMR was performed using a Bruker AVANCE NEO 500 MHz NMR spectrometer, and 1H NMR data were processed using Bruker TopSpin software. To ensure quantitative accuracy, each spectrum was baseline-corrected and phase-corrected using software commands. To determine the ratios of anomeric protons in each spectrum, peaks of interest were integrated using software commands, which generated a value between 0 and 1. Higher integral values indicated higher ratios of the glycosylation states represented by the proton signals.

## Data Availability

All sequenced genomes are available in the NCBI database under BioProject PRJNA1285472.

## SUPPLEMENTAL MATERIAL

Fig. S1 aac.00396-25-s0001.tif *mecA* and *mecR1* expression in FBS and FBS contain 1×MIC cefazolin.

Fig. S2 aac.00396-25-s0002.tif NARSA library *clp* and *rpo* mutants have comparable RpoB protein expression levels.

Supplemental material aac.00396-25-s0003.docx Supplemental figure legends.

ASM does not own the copyrights to Supplemental Material that may be linked to, or accessed through, an article. The authors have granted ASM a non-exclusive, world-wide license to publish the Supplemental Material files. Please contact the corresponding author directly for reuse.
