# Supplementary figures and images for: Methicillin-resistant Staphylococcus aureus has phenotypic variation in mecA expression that alters antibiotic sensitivity
Source: Antimicrob Agents Chemother. 2026 Feb 12;70(3):e00396-25. doi: 10.1128/aac.00396-25 (PMC12959143; doi:10.1128/aac.00396-25)

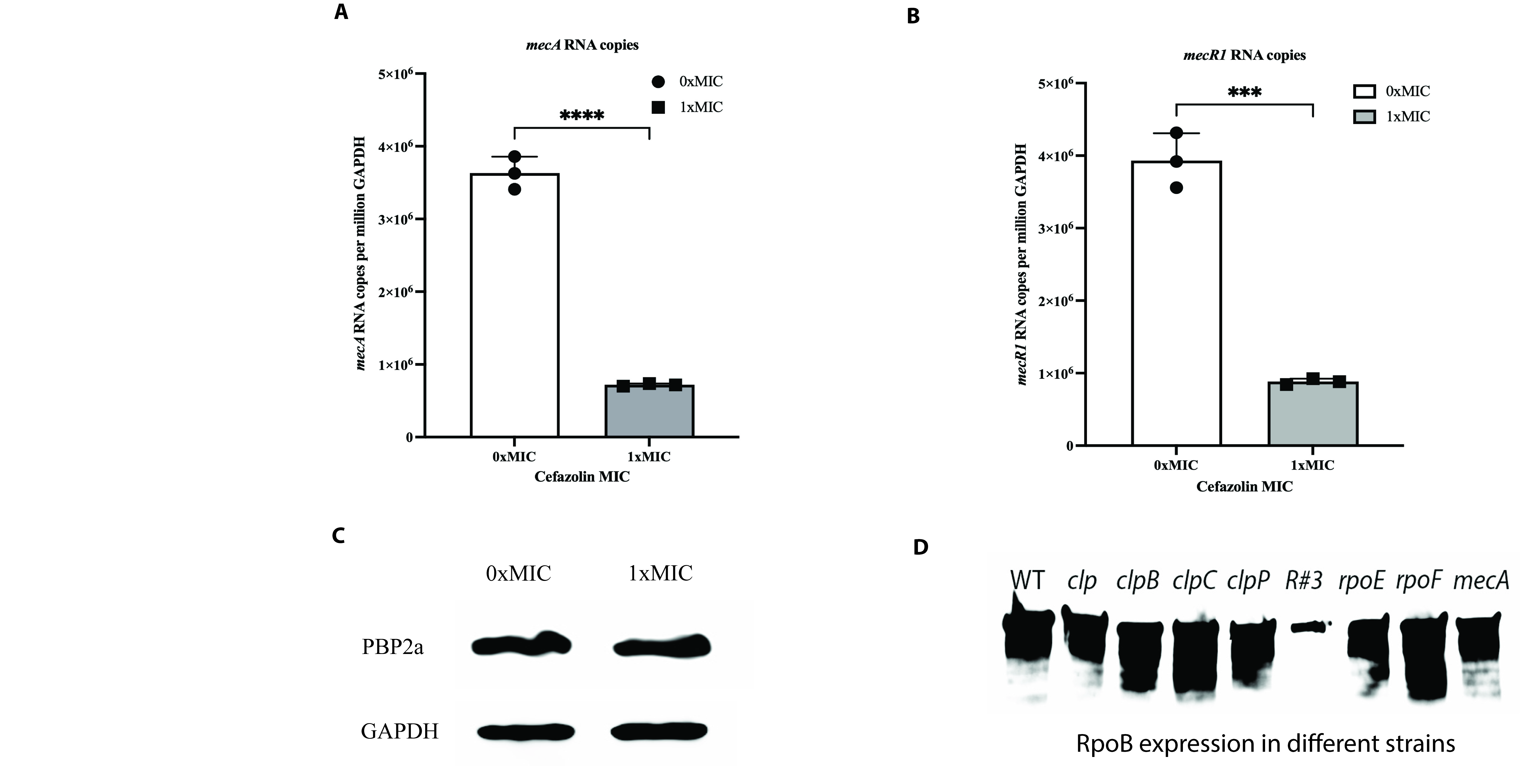

Supplement: Fig. S1 — mecA and mecR1 expression in FBS and FBS contain 1×MIC cefazolin. [file aac.00396-25-s0001.tif]

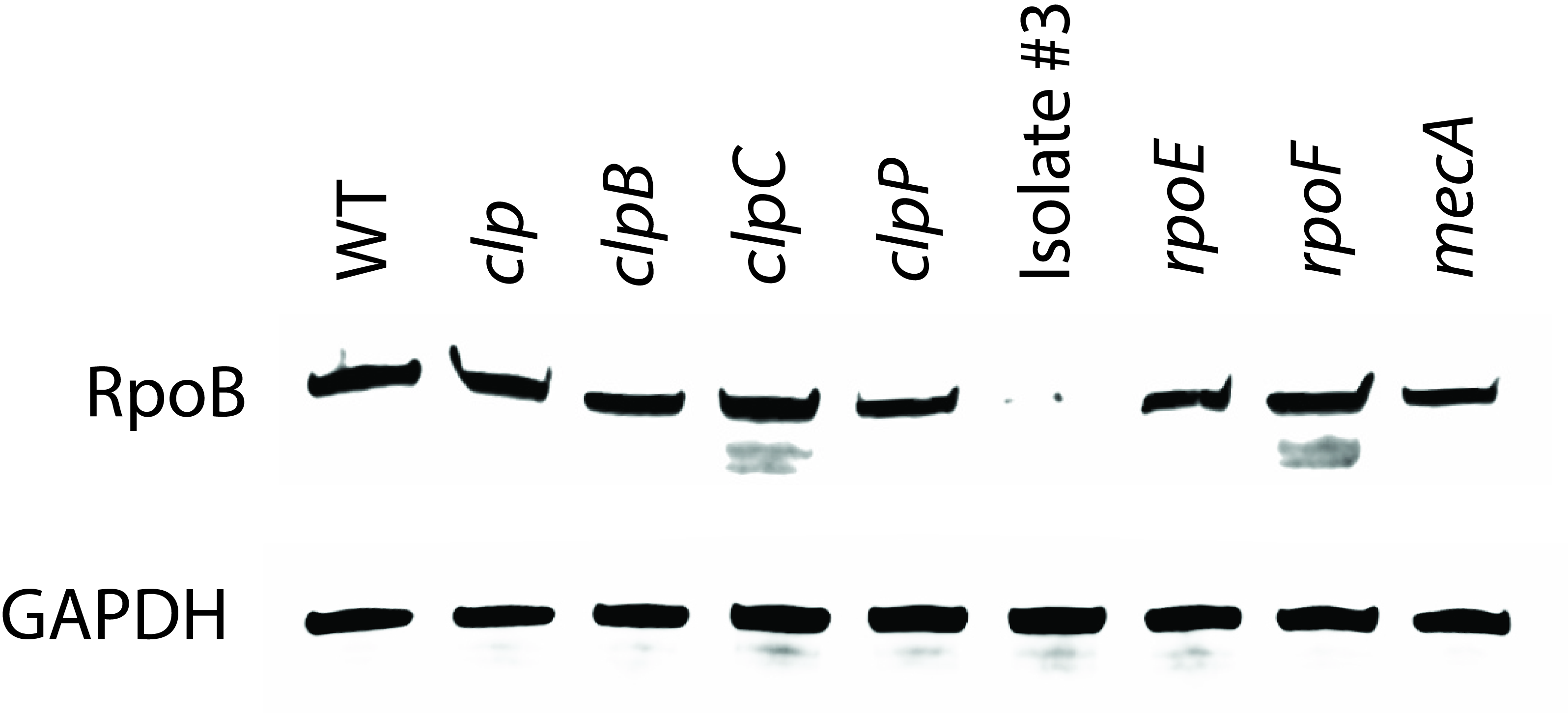

Supplement: Fig. S2 — NARSA library clp and rpo mutants have comparable RpoB protein expression levels. [file aac.00396-25-s0002.tif]
