# Supplementary material for: Methicillin-resistant Staphylococcus aureus has phenotypic variation in mecA expression that alters antibiotic sensitivity
Source: Antimicrob Agents Chemother. 2026 Feb 12;70(3):e00396-25. doi: 10.1128/aac.00396-25 (PMC12959143; doi:10.1128/aac.00396-25)
Supplement: Supplemental material — Supplemental figure legends. [file aac.00396-25-s0003.docx]

**Genomic bacterial DNA isolation**

MasterPure™ Gram Positive DNA Purification Kit (Lucigen Corp., USA) was used to isolate genomic DNA from the *Staphylococcus aureus* samples. Clinical *S. aureus* stock samples stored at -80 were streaked out on TSA plate and then incubated overnight at 37 ̊C. A single colony from TSA plate was picked then inoculated in 4 mL TSB medium then grown overnight at 37 ̊C in a 250 rpm shaker. 1.5 mL of the overnight grown bacterial culture was pelleted. 150 μL of TE Buffer was added and then vortexed to resuspend the cell pellet. 1 μL of Ready-Lyse Lysozyme and 5 μL of lysostaphin (5 mg/mL) were added to each resuspended pellet of bacteria. The samples were incubated at 37 ̊C until bacterial cell wall was destroyed. 150 μL of Proteinase K/Gram Positive Lysis Solution was added to the sample and then mixed thoroughly. Samples were incubated at 65 ̊C for 15 minutes, vortexing briefly every 5 minutes. The samples were cooled to 37 ̊C. Samples were placed on ice for 5 minutes and then 175 μL of MPC Protein Precipitation Reagent was added to 300 μL of lysed sample and then vortexed vigorously for 10 seconds. The debris was pelleted by centrifugation at 4 ̊C for 10 minutes at 12,000 x g. The supernatant was transferred to a clean microcentrifuge tube and the pellet was discarded. 1 μL of RNase A (5 μg/μL) was added to each sample and mixed thoroughly. Samples were then incubated at 37 ̊C for 10 minutes. 500 μL of isopropanol was added to the recovered supernatant. The tubes were inverted 30-40 times. The DNA was pelleted by centrifugation at 4 ̊C for 10 minutes at 12,000 x g. A pipet tip was used to remove the isopropanol without dislodging the DNA pellet. The pellet was rinsed with 70% ethanol. The DNA was resuspended in 100 μL of TE Buffer. The genomic DNA was used as template in following PCR reactions and sequencing.

**Markerless *rpoB* gene deletion using pKFT Vector**

Followed the protocol from Dr. Fuminori Kato. Briefly, JE2 *rpoB* upstream region was amplified by JE2-rpoB-5-F (5′-GTAgtcgacGGTTCAGATGTTCTTGTTCAAACT-3′) and JE2-rpoB-5-R (5′- ATTtctagaAAACCTTCTCTTAGGAACCACTC-3′) and cloned into pKFT vector SalI and XbaI sites.

JE2 *rpoB* downstream region was amplified by JE2-rpoB-3-F (5′- AATtctagaGACAGACGTTGATGACGATGA-3′) and JE2-rpoB-3-R (5′- TGGggatccTTCTGGTGGGATGATTGGAAG-3′) and cloned into pKFT vector containg *rpoB* upstream region generated before at XbaI and BamHI sites to create the allelic replacement vector pKFT-ropB. pFK-rpoB (containing regions upstream and downstream of the *rpoB* gene) was first transformed into DNA restriction system-deficient *S. aureus* RN4220, then a modified plasmid was isolated and electroporated into JE2 strain. Transformants were selected at 30 ̊C on TSB plates containing tetracycline. Then, single colony transformants were grown at 30 ̊C in 4 ml TSB containing tetracycline at 250 rpm shaking. Integration of the plasmid into the chromosome by a single crossover event was achieved by incubation at 42 ̊C on TSB plates containing tetracycline. Correct homologous recombination of the target region was verified by PCR using primer set of pUC-UV (5’-CGACGTTGTAAAACGACGGCCAGT-3’, plasmid) and JE2-rpoB-up-F (5’- CTTCACACGATGGTCTTGTATCT-3’, chromosome) or pUC-RV (5’- CACAGGAAACAGCTATGACCATG-3’, plasmid) and JE2-rpoB-dn-R (5’- CGACCACGACGACCATTATC-3’, chromosome). Then, the correct integrants were grown at 25 ̊C overnight with shaking in 10 ml TSB without any antibiotics; repeat twice. Then the cells were serially diluted and plated on TSB plates at 42 ̊C. The excision of the plasmid region in the chromosome by a double-crossover event was screened for tetracycline-sensitive colonies by replica-plating candidates on TSB plates versus TSB plates containing tetracycline (3 μg/ml). The integrants were incubated at 37 ̊C overnight. Then, the markerless deletion mutants were screened by PCR using primers JE2-rpoB-5-F and JE2-rpoB-3-R from tetracycline-sensitive colonies. The strain with *rpoB* deletion will be screened by PCR and confirmed by genomic DNA sequencing.

**Figure legends**:

Figure S1. *mecA* and *mecR1*expression in FBS and FBS contain 1xMIC cefazolin. Quantitative real-time PCR was performed to test mRNA levels and Western blotting were performed to test protein levels. (A) *mecA* mRNA expression of JE2 cultured in FBS and FBS contain 1xMIC cefazolin. (B) *mecR1* mRNA expression of JE2 cultured in FBS and FBS contain 1xMIC cefazolin. (C) PBP2a expression of JE2 cultured in FBS and FBS contain 1xMIC cefazolin. (D) RpoB expression of JE2 in *clp* and *rpo* mutants (Higher exposure). Comparisons were performed using unpaired *t-test*. (***p<0.001, ****p<0.0001)

Figure S2. NARSA library *clp* and *rpo* mutants have comparable RpoB protein expression levels. Western blot analysis of RpoB was performed in NARSA mutant strains *clp, clpB, clpB, clpC, clpP, rpoE, rpoF,* and *mecA* in FBS. (*mecA* is a *mecA* gene mutation strain from NARSA library as a control here). (The ECL had a very strong signal. Using a 15 second exposure, the RpoB band of #3 isolate is visible (Figure S1D). Given the strength of signal of the other bands, a shorter exposure time needed to be used to more clearly observe the other bands, but this made isolate #3 RpoB almost invisible).
